# Everybody's Page

**Published:** 1889-06-08

**Authors:** 


### Everybody's Page.

In Kensington and Bayswater alone sixty-one beggars were apprehended between December 1 and January 14 last.

Dr. Cheyne is of opinion that epilepsy is as certain a manifestation of a scrofulous constitution as tubercular consumption. It is often connected with some malformation or lack of symmetry about the head.

Till the sixteenth century alcohol was used only as a medicine. It was as a drug and not as a beverage that it became known as *aqua vitæ*—the water of life, from its great restorative powers. In 1581 it was first introduced as part of the food allowance of the English army in the Netherlands.

The advent of the hot weather has brought the usual anxiety about hydrophobia, and it is probable that an edict condemning all dogs to be muzzled will soon be promulgated. The dogs dislike this, naturally; but, like other creatures, the innocent among them must bear a certain amount of restraint because of the mischief their guilty brethren may do.

Many people in towns sleep with their windows shut, even in summer, "because," they say, "the air is not pure." But surely, if the outside air is not as pure as that of sea or mountain, the supply of that same air which they have stored up in their bedroom, and inhale and expire over and over again during the night, is very much less so.

It is not intellectual work that injures the brain, but emotional excitement. Most men can stand the severest thought and study of which their brains are capable, and be none the worse for it; for neither thought nor study interfere with the recuperative influence of sleep. It is ambition, anxiety, and disappointment, the hopes and fears, the loves and hates of our lives, that wear out our nervous system and endanger the balance of the brain.

Mr. Isaac Frankenberg, of Manchester, himself an employer of labour, has made a valuable suggestion in his evidence before the Commission that is enquiring into the sweating system. He wishes that every workroom should contain a placard stating the amount of cubic air space in it, and the number of workpeople it ought to contain. This is to be put up in a place where the workmen can see it, so that they may be able to tell at once when there is overcrowding. Often they submit to insanitary conditions of all sorts, this included, from sheer ignorance of their danger.

What will be the effect on the cynical mind of the House of Lords sitting in judgment on a dress improver? It was a question of whether a certain wire bustle was so different from all other bustles that it could claim to be protected by a patent. Lord Herschell thought it had a right to such protection, while Lord Fitzgerald was of opinion that the bustle was an old invention, as old indeed as the time when "the art of the dressmaker was called in to disfigure nature," and held that one bustle was very like another. But the Lord Chancellor—think of it, ladies, the Lord Chancellor giving judgment on dress improvers!—held that the particular specimen of braided wire was unique and deserved protection. If anything could give the *coup de grâce* to this moribund fashion, surely it would be the picture of five learned gentlemen, lawyers and peers, sitting in judgment on a bustle.

"What a difference it makes," said a desponding medico, as he looked at a letter he had just received, "whether you put Dr. before a name or after it."

It is very bad economy to read in the dark. The saving in gas or candle light is more than counterbalanced by the injury to the sight.

At the time of the Reformation the average life of man was twenty-one and a quarter. In the early part of this ceutury it was rather more than forty and a half, and certainly has not lessened since. That means that as many people live to seventy now as lived to forty, three hundred years ago.

Dr. Imlach, of Liverpool, who has given a good deal of attention to the subject, has come to the conclusion that consumption can be transmitted from cows to human beings through milk. His experiments prove that guinea-pigs, rabbits, and monkeys, fed on the milk of tuberculous cows develop tubercular disease.

Surgeon Parry, of the Indian Medical Department, says that he saw the jet black hair of a rebel Sepoy turn grey in half an hour, while he was under examination and half mad with fear. Another strange story is that of a gentleman who left home on his wedding tour with dark hair. When he came back, a month later, his hair, his beard, and even his eyebrows, had become snow-white. This seems to reflect somewhat severely on his wife's temper. Was it fright that turned him grey also?

Occasionally we are shocked to hear of suicide on the part of a child, and are, perhaps, too ready to assume that it is due to some cruelty, or, at least, unwarranted severity on the part of parents or guardians. As a matter of fact, the immediate cause of the reckless act is often slight enough—a childish grievance that would scarcely cause another a fit of crying; and the real cause will be found to be an inherited tendency to melancholia. In many cases suicidal mania may be traced back to ancestors; but, of course, if their attempts at self-slaughter have not been successful, it is unlikely that they will be remembered by the next generation.

An American journal gives an interesting account of an "arbeiter colonie," a sort of experiment in home colonisation, that has been started near Berlin. In 1882 some young men began providing a Sunday breakfast to a few unemployed workmen. They were struck with the frequency with which their guests declared that they would rather have work than charity, and were therefore induced to give them a chance. They obtained a house and a plot of ground, established their *protégés* in their various trades—carpentry, brush and mat making, and the like—sold their goods for them, and paid them in money. The workers were expected then to pay for their food and lodging, which was provided on the premises. There are now a reading-room, a dancing-room, a hall for religious and other meetings, besides baths and other comforts, and the colony seems to go on both successfully and happily—chiefly, perhaps, because the originators of the scheme take a friendly and practical interest in it.

